# Association between retinal microvasculature and optic disc alterations in high myopia

**DOI:** 10.1038/s41433-019-0438-7

**Published:** 2019-04-24

**Authors:** Jiangnan He, Qiuying Chen, Yao Yin, Hongfeng Zhou, Ying Fan, Jianfeng Zhu, Haidong Zou, Xun Xu

**Affiliations:** 1 Shanghai Eye Disease Prevention and Treatment Center, Shanghai Eye Hospital, Shanghai, China; 2 Shanghai General Hospital, Shanghai Jiaotong University School of Medicine, Shanghai, China; 3 Shanghai Jiaotong University School of Medicine, Shanghai, China; 4 Shanghai Engineering Center for Visual Science and Photo medicine, Shanghai Jiaotong University School of Medicine, Shanghai, China; 5 School Hospital, Shanghai University, Shanghai, China

**Keywords:** Retinal diseases, Refractive errors

## Abstract

**Purpose:**

This study aimed to explore the characteristics of retinal perfusion and its associations with high myopia.

**Methods:**

A total of 760 participants were included. Peripapillary radial peripapillary capillary perfusion, foveal avascular zone, and parafoveal perfusion were measured using optical coherence tomography angiography (OCTA). Tilted disc ratio and parapapillary atrophy were determined using swept-source optical coherence tomography.

**Results:**

A total of 760 young healthy participants with myopic eyes were included in the analysis. The mean axial length and titled disc ratio were 26.43 ± 1.14 and 0.76 ± 0.08 mm in the high-myopia group and 24.79 ± 0.75 and 0.80 ± 0.09 mm in the control group, respectively. The high-myopia group exhibited significantly larger parapapillary atrophy, lower tilted disc ratio, lower radial peripapillary capillary vessel density, larger area of foveal avascular zone, and lower deep parafoveal vessel density. In the multivariate analysis, titled disc ratio significantly correlated with radial peripapillary capillary vessel density (*P* = 0.0134), larger foveal avascular zone (*P* = 0.0062), and lower deep parafoveal vessel density (*P* < 0.0001).

**Conclusions:**

Reduced radial peripapillary capillary and deep parafoveal vessel density and enlarged area of foveal avascular zone were observed in high myopia. Tilted disc ratio correlated with retinal perfusion.

## Introduction

Myopia is the leading cause of correctable visual impairment even in younger persons in the world [1]. It is estimated that by 2050, 5 billion and 1 billion people will be affected by myopia and high myopia (HM), respectively. The continuously increasing prevalence of myopia, especially in Asian populations, highlights the importance of taking action to prevent the progression of myopia, ocular complications, and vision loss resulting from HM [2–4]. HM can lead to myopic retinopathy, which is significantly associated with retinal vessel morphologic alterations [5, 6]. Therefore, retinal perfusion in HM has been an important issue for several decades. It may provide a critical clue for understanding the pathophysiology of HM-related diseases. Reduced retinal vessel density or blood flow in patients with HM has already been reported using different techniques [7–9].

Optical coherence tomography angiography (OCTA) is a recently introduced noninvasive vascular imaging technology that uses intrinsic motion contrast to detect blood flow within a microcirculatory network. The recent development of split-spectrum amplitude-decorrelation angiography (SSADA) has made the quantitative observation of retinal perfusion possible [10–14]. OCTA with SSADA has been demonstrated to have high intra-visit repeatability and inter-visit reproducibility when used to assess peripapillary and macular vessel density [12–14].

Based on this background, this study aimed to investigate the early characteristics of peripapillary and macular retinal perfusion and ocular variables in HM. It focused on the association between vascular parameters and tilted disc ratio in a myopia population.

## Methods

### Setting and participants

This cross-sectional population-based study was approved by the ethics committee of Shanghai General Hospital, Shanghai Jiao Tong University, Shanghai, China, and followed the tenets of the Declaration of Helsinki. The participants understood the study protocol and signed informed consents were obtained from all of them. The study was registered at www.clinicaltrials.gov (no. NCT03446300).

The participants were randomly selected from students of the Shanghai University in October 2016, who were seriously affected by HM. The students’ systolic blood pressure (SBP) and diastolic blood pressure (DBP), pulse rate, height, and weight were measured. The BP amplitude was calculated using the following formula: SBP − DBP. The mean arterial pressure (MAP) was calculated using the following formula: MAP = DBP + 0.42 (SBP − DBP) [15–17]. The body mass index (BMI) was calculated using the following formula: weight (kg)/[height (m)]^2^. A detailed medical history was recorded for each student. All participants underwent comprehensive ophthalmic examinations, including refractive error assessment using an autorefractor machine (model KR-8900; Topcon, Tokyo, Japan), measurement of best-corrected visual acuity (BCVA) and intraocular pressure (IOP, Full Auto Tonometer TX-F; Topcon), slit-lamp biomicroscopy, and color fundus examination. Central corneal thickness (CCT), lens thickness (LT), anterior chamber depth (ACD), and axial length (AL) were measured using optical low-coherence reflectometry (Aladdin; Topcon, Tokyo, Japan). Subjective refraction was performed by a trained optometrist for all of the participants. Spherical equivalent (SE) was calculated as the sphere plus half a cylinder. The BCVA was converted into the logarithm of minimal angle resolution (logMAR).

The inclusion criteria were as follows: age between 18 and 25 years; SER < 0.5 D; BCVA ≥ 20/25, namely logMAR ≤ 0.1; IOP ≤ 21 mm Hg; normal anterior chamber angles; normal optic nerve head without glaucomatous changes, such as narrowing of the neuroretinal rim, increased cup–disc ratio, and peripapillary hemorrhage; and no retinal nerve fiber layer abnormalities on red-free fundus photography. Participants with a history of ocular or systemic diseases, including congenital cataract and glaucoma, hypertension, and diabetes; previous intraocular or refractive surgery; and other evidence of retinal pathology were further excluded. Only the right eye of each patient was selected for statistical analyses.

### OCTA acquisition and processing

The OCTA scans were made using a spectral-domain system (RTVue-XR Avanti; Optovue, CA, USA). Optic disc (scan size of 6 × 6 mm^2^) and parafoveal (scan size of 6 × 6 mm^2^) OCTA scans were acquired in two consecutive B-scans at 304 raster positions, and each B-scan consisted of 304 A-scans.

**Fig. 1:**
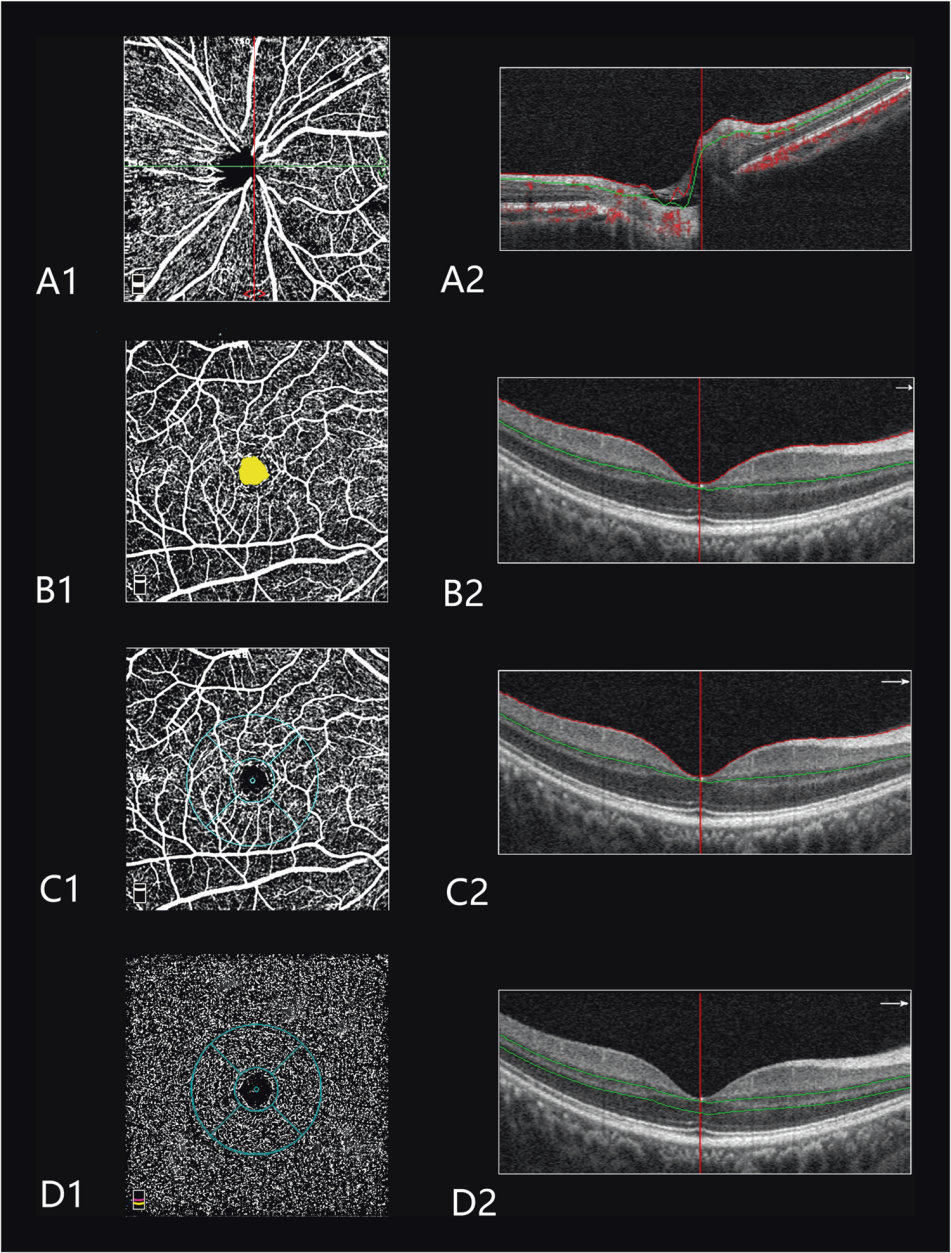
Peripapillary and macular perfusion were measured using optical coherence tomography angiography (OCTA) scans of the emmetropic eye. **A1** Peripapillary angioflow density map of the radial peripapillary capillary layer. **A2** The boundaries used for segmentation are indicated between the red and green lines on the cross-sectional OCTA reflectance. **B1** Foveal avascular zone of the superficial layer. **B2** The boundaries used for segmentation are indicated between the red and green lines on the cross-sectional OCTA reflectance. **C1** Macular angioflow density map in the superficial layer. The sectors for measurements include the following: temporal, superior, nasal, and inferior. **C2** The boundaries used for segmentation are indicated between the red and green lines on the cross-sectional OCTA reflectance. **D1** Macular angioflow density map in the deep layer. The sectors for measurements include the following: temporal, superior, nasal, and inferior. **D2** The boundaries used for segmentation are indicated between two green lines on the cross-sectional OCTA reflectance

The angioflow vessel density was expressed as a percentage of the measured area and measured using the commercially unreleased software (version 2016.1.0.23). In this study, the peripapillary vessel density was measured in radial peripapillary capillary (RPC) layers (Fig. 1A1, A2). The foveal avascular zone (FAZ) area was outlined and automatically measured using the contained software (Fig. 1B1, B2). The parafoveal vessel density was measured in two layers, including the superficial and deep layers (Fig. 1C1, C2, D1, D2). Littman and the modified Bennett formulas were used to correct the area of FAZ induced by AL variation [18]. The exclusion criteria applied to the OCTA scans were a signal strength index <40 and images with segmentation errors or residual motion artifacts.

### Swept-source optical coherence tomography imaging

The peripapillary and macular retinal nerve fiber layer thickness (pRNFLT and mRNFLT), macular ganglion cell layer thickness (pGCLT and mGCLT), peripapillary and macular retinal thickness, and peripapillary and macular choroidal thickness were measured using the Swept-source optical coherence tomography (SS-OCT; model DRI OCT-1 Atlantis; Topcon). The global average foveal thicknesses of the choroid, retina, GCL, and RNFL were calculated. The circle placement was manually adjusted if necessary. All measurements were conducted by a single technician experienced in taking SS-OCT images. Images with signal strength index ≤60 were excluded from the statistical analysis.

### Assessment of optic disc tilt and parapapillary atrophy area

Retinal photographs centered on the macular and optic discs were acquired from the same SS-OCT. The optic disc tilt and the parapapillary atrophy (PPA) area were calculated from these photographs using the ImageJ version 1.60 software (National Institutes of Health, MD, USA) by two independent, blinded, well-trained observers (QC and YY) [19–21]. Average data were used for the final analysis. The magnification was corrected for each AL applying the Littmann’s formula [18].

The association between AL and vascular change was stronger than the association between SE and vascular change. All eyes were divided into four groups based on the degree of AL: the emmetropia (EM) group with AL between 23 and 24 mm; the mild-myopia (MIM) group with AL between 24 and 25 mm; the moderate-myopia (MOM) group with AL between 25 and 26 mm; and the HM group with AL ≥ 26 mm.

### Statistical analysis

Data analyses were performed on a computer equipped with statistical software (Statistical Analysis System, version 9.3; the SAS Institute, NC, USA). Demographic and ocular characteristics were shown as counts or proportions for categorical data and as mean ± standard deviation for continuous data. The analysis of variance (ANOVA) test was performed to detect differences in demographic and ocular parameters and the thickness of each layer between the four groups as appropriate. The univariate analysis was used to investigate the relationship between peripapillary or macular perfusion and ocular parameters adjusted for gender and age. Parameters with *P* values < 0.05 in the univariate analysis were included in the stepwise multivariate models. Standardized regression coefficients, adjusted coefficients of determination (*R*^2^), and variance inflation factor were calculated from the multivariable linear regression models after excluding variables that showed multicollinearity. For the tilted disc ratio, an exploratory analysis involving the subgrouping of eyes with HM by quartiles of tilted disc ratio was performed to assess for potential differential effects of severe (lower quartile, Q1) versus mild (upper quartile, Q4) tilted ratio in the HM group compared with controls (including EM, MIM, and MOM groups).

## Results

Among the 828 students enrolled in the study, 5 were excluded due to AL <23 mm, 6 were excluded due to BCVA lower than 20/25, 12 were excluded because of IOP higher than 21 mm Hg, and another 45 were excluded owing to poor OCTA images. Finally, 760 (91.79%) students were included in the analysis. Based on the International Photographic Classification and Grading System for myopic maculopathy (META-PM), 3, 571, and 186 of 760 eyes had category 2 (0.39%, diffuse chorioretinal atrophy), category 1 (75.13%, tessellated fundus), and category 0 (24.47%, no myopic retinal changes) myopic maculopathy, respectively.

Among all 760 participants, 85 had EM (11.18%), 211 had MIM (27.76%), 243 had MOM (31.97%) and 221 had HM (29.08%). The general characteristics of the enrolled participants are listed in Table 1. No significant differences in age, BMI, SBP, DBP, BP, MAP, PR, and CCT were found among the four groups. The HM group included a few female participants (*P* = 0.0004). However, the eyes with HM had a lower SE, worse BCVA, deeper ACD, and thinner LT.

**Table 1:** Demographic and ocular characteristics of the study population

Variable	EM (*N* = 85)	MIM (*N* *=* 211)	MOM (*N* = 243)	HM (*N* = 221)	*P* value	Post hoc
Age, year	20.19 ± 2.53	19.88 ± 3.03	19.82 ± 2.36	20.03 ± 2.58	0.2102	/
Female, *n* (%)	52 (61.18)	131 (62.09)	126 (51.85)	101 (45.70)	0.0004	MIM > HM
BMI	20.79 ± 2.79	20.62 ± 2.93	21.01 ± 3.20	20.67 ± 2.90	0.5023	/
SBP, mm Hg	121.38 ± 15.49	119.74 ± 16.42	121.88 ± 15.91	123.40 ± 15.96	0.1280	/
DBP, mm Hg	73.89 ± 10.06	72.05 ± 11.87	72.39 ± 9.67	72.95 ± 9.45	0.5137	/
BP amplitude, mm Hg	47.48 ± 10.99	47.69 ± 12.63	49.49 ± 11.87	50.44 ± 12.07	0.0610	/
MAP, mm Hg	93.84 ± 11.40	92.08 ± 12.49	93.18 ± 11.24	94.14 ± 11.10	0.2978	/
PR, bpm	75.30 ± 9.70	75.29 ± 9.97	74.71 ± 11.01	74.45 ± 9.75	0.8120	/
SE, D	–1.80 ± 1.47	–2.89 ± 1.81	–4.39 ± 2.04	–5.77 ± 2.95	<0.0001	EM > MIM > MOM > HM
AL, mm	23.63 ± 0.27	24.54 ± 0.29	25.42 ± 0.48	26.43 ± 1.14	<0.0001	EM < MIM < MOM < HM
BCVA, logMAR	0.01 ± 0.03	0.02 ± 0.04	0.03 ± 0.08	0.04 ± 0.09	0.0029	MIM < HM; EM < HM
IOP, mm Hg	13.95 ± 3.01	14.20 ± 2.96	14.05 ± 3.09	14.22 ± 3.04	0.8528	/
ACD, mm	3.59 ± 0.24	3.66 ± 0.22	3.75 ± 0.20	3.78 ± 0.26	<0.0001	EM < MOM/HM; MIM < MOM/HM;
CCT, μm	539.71 ± 39.80	539.89 ± 35.02	536.34 ± 36.26	533.94 ± 36.99	0.3327	/
LT, mm	3.58 ± 0.23	3.53 ± 0.24	3.50 ± 0.18	3.49 ± 0.21	0.0023	EM < MOM; EM < HM

Factors with statistical significance are shown in boldface. Numbers displayed are mean ± standar deviation. All values were calculated using the one-way analysis of variance, except the values in bold, which were calculated using the *χ*^2^ test. Tukey multiple comparisons were performed among the four axial length groups

*ACD* anterior chamber depth, *AL* axial length, *BCVA* best-corrected visual acuity, *BMI* body mass index, *BP* blood pressure, *CCT* central corneal thickness, *CR* corneal radius, *D* diopters, *DBP* diastolic blood pressure, *EM* emmetropia, *HM* high myopia, *IOP* intraocular pressure, *LT* lens thickness, *MIM* mild myopia, *MOM* moderate myopia, *MAP* mean arterial pressure, *PR* pulse rate, *SBP* systolic blood pressure, *SE* spherical equivalent

The topographic structural and perfusion parameters in the peripapillary area of the participants and multiple comparisons are shown in Table 2. No significant difference in average pRNFLT was observed among the four groups. The average pReT, pGCLT + pRNFLT, and pChT were lower in eyes with HM than in eyes with EM, MIM, and MOM (*P* < 0.0001, ANOVA). The area of PPA was significantly larger in eyes with HM than in eyes with EM, MIM, and MOM. The tilted disc ratio was significantly lower in eyes with HM than in eyes with EM, MIM, and MOM. The RPC vessel density was significantly lower in eyes with HM than in eyes with EM, MIM, and MOM (*P* < 0.0001, ANOVA).

**Table 2:** Structural and perfusion parameters in the peripapillary and macular region of the participants between different myopia groups

Variable	EM (*N* = 85)	MIM (*N* = 211)	MOM (*N* = 243)	HM (*N* = 221)	*P* value	Post hoc
pReT, µm	290.71 ± 16.16	285.87 ± 13.42	281.53 ± 14.07	278.21 ± 15.43	<0.0001	EM > MOM/HM; MIM > MOM/HM
pRNFLT, µm	102.19 ± 10.05	102.18 ± 7.14	102.26 ± 8.03	102.93 ± 9.13	0.8366	/
pGCLT + pRNFL, µm	146.21 ± 11.48	144.30 ± 8.46	142.74 ± 9.04	142.30 ± 10.34	0.0137	EM > MOM; EM > HM
pChT, µm	154.39 ± 42.71	145.43 ± 41.40	137.79 ± 41.90	123.25 ± 40.01	<0.0001	EM > MOM > HM; MIM > HM
Area of PPA,mm^2^	0.09 ± 0.08	0.14 ± 0.10	0.19 ± 0.14	0.26 ± 0.17	<0.0001	EM < MIM < MOM < HM
Tilted disc ratio	0.83 ± 0.08	0.80 ± 0.09	0.78 ± 0.09	0.76 ± 0.08	<0.0001	EM > MIM > HM; EM > MOM
RPC vessel density,%	45.02 ± 3.76	44.40 ± 3.62	43.13 ± 3.99	42.98 ± 4.42	<0.0001	EM > MOM/HM; MIM > MOM/HM
mReT, µm	281.59 ± 12.44	277.61 ± 11.99	275.95 ± 10.95	274.26 ± 12.74	<0.0001	EM > MOM; MIM > HM;EM > HM
mRNFLT, µm	40.57 ± 4.28	40.56 ± 4.69	40.61 ± 5.46	39.98 ± 5.39	0.5402	/
mGCLT + RNFL, µm	111.39 ± 6.57	110.07 ± 5.82	109.57 ± 5.85	109.21 ± 6.08	0.0684	/
mChT, µm	249.54 ± 52.95	233.15 ± 54.11	212.46 ± 58.40	191.73 ± 56.24	<0.0001	EM/MIM > MOM > HM
Foveal ReT, µm	230.38 ± 17.63	226.88 ± 18.36	227.16 ± 16.98	227.53 ± 19.14	0.4807	/
Foveal RNFLT, µm	7.48 ± 2.98	8.04 ± 3.86	8.19 ± 3.47	7.36 ± 3.48	0.0462	/
Foveal GCLT + RNFLT, µm	52.17 ± 7.93	51.85 ± 9.71	51.69 ± 7.80	50.41 ± 8.91	0.3151	/
Foveal ChT, µm	264.19 ± 60.69	245.24 ± 64.14	216.26 ± 68.42	190.67 ± 64.88	<0.0001	EM/MIM > MOM > HM
Area of FAZ, mm^2^	0.35 ± 0.10	0.36 ± 0.11	0.37 ± 0.11	0.39 ± 0.13	0.0052	EM/MIM < HMfoveal
Superficial parafoveal vessel density, %	48.80 ± 3.62	48.50 ± 3.94	48.05 ± 3.98	47.59 ± 3.88	0.0329	/
Deep parafoveal Vessel density, %	60.52 ± 3.16	59.98 ± 3.78	58.93 ± 3.96	57.49 ± 4.96	<0.0001	EM/MIM > MOM > HM

Numbers displayed are mean ± standard deviation. All values were calculated using the one-way analysis of variance. Tukey multiple comparisons were performed among the four axial length groups

*ChT* choroidal thickness, *EM* emmetropia, *FAZ* foveal avascular zone, *GCLT* ganglion cell layer thickness, *HM* high myopia, *mChT* macular choroidal thickness, *mGCLT* macular ganglion cell layer thickness, *MIM* mild myopia, *MOM* moderate myopia, *mRNFLT* macular retinal nerve fiber layer thickness, *mRT* macular retinal thickness, *pChT* peripapillary choroidal thickness, *pGCLT* peripapillary ganglion cell layer thickness, *PPA* parapapillary atrophy, *pRNFLT* peripapillary retinal nerve fiber layer thickness, *pReT* peripapillary retinal thickness, *ReT* retinal thickness, *RPC* radial peripapillary capillary, *RNFLT* retinal nerve fiber layer thickness

The topographic characteristics of structural and perfusion parameters in the macular area of the participants and multiple comparisons are listed in Table 2. No significant differences in mRNFLT, mGCLT + mRNFLT, foveal retinal thickness, foveal RNFLT, foveal GCLT + RNFLT, and superficial parafoveal vessel density were found among the four groups. The average mReT, mChT, and foveal choroidal thickness were lower in eyes with HM than in eyes with EM, MIM, and MOM. The FAZ area in the superficial layer was lower in eyes with HM than in eyes with EM and MIM (*P* *=* 0.0052, ANOVA). The deep parafoveal vessel density was lower in eyes with HM than in eyes with EM, MIM, and MOM (*P* *<* 0.0001, ANOVA).

Next, univariate regression analyses adjusted for age and sex were conducted to investigate the association between RPC vessel density, area of FAZ, and ocular variables in all eyes. Variables with a significance of *P* < 0.05 in the univariable analysis are shown in Table 3. In the univariate analysis, the RPC vessel density significantly correlated with AL, tilted disc ratio, area of PPA, SE, pRNFLT, and pGCLT + RNFLT, whereas FAZ significantly correlated with AL, tilted disc ratio, area of PPA, SE, CCT, foveal ReT, foveal ChT, foveal GCLT + RNFLT, and foveal RNFLT (Table 3). Variables with a significance of *P* < 0.05 were included in the stepwise multivariate models, except for SE that had a stronger correlation with AL. The multivariable analysis revealed that AL (*P* < 0.0001), tilted disc ratio (*P* = 0.0134), pReT (*P* = 0.0125), and pRNFLT (*P* < 0.0001) were associated with the RPC vessel density. Meanwhile, AL (*P* = 0.0171), tilted disc ratio (*P* = 0.0062), foveal ReT (*P* = 0.0081), foveal GCLT + RNFLT (*P* < 0.0001), and foveal RNFLT (*P* = 0.0128) were associated with the area of FAZ (Table 3).

**Table 3:** Linear regression analysis with RPC, superficial parafoveal, deep parafoveal vessel density and FAZ as dependent variables

Variables	Coefficient	*R* ^2^	*P* value	Coefficient	VIF	*P* value
RPC vessel density				*R*^2^ = 0.1646		
AL, mm	−0.26	0.0949	<0.0001	−0.29	1.48	<0.0001
Tilted disc ratio	0.19	0.0644	<0.0001	0.11	1.17	0.0134
Area of PPA, mm^2^	−0.18	0.0577	<0.0001			
SE, D	0.19	0.0624	<0.0001	–	–	–
pReT, µm	0.15	0.0561	0.0003	−0.20	3.74	0.0125
pRNFLT, µm	0.14	0.0530	0.0008	0.29	2.42	<0.0001
pGCLT + RNFLT, µm	0.20	0.0713	<0.0001			
Area of FAZ, mm^2^				*R*^2^ = 0.5639		
AL, mm	0.22	0.0569	<0.0001	0.07	1.10	0.0171
Tilted disc ratio	−0.14	0.0307	0.0005	−0.09	1.13	0.0062
Area of PPA, mm^2^	0.21	0.0544	<0.0001			
SE, D	−0.19	0.0457	<0.0001	–	–	–
CCT, μm	−0.09	0.0184	0.0249			
Foveal ReT, µm	−0.64	0.4053	0.0000	−0.15	3.50	0.0081
Foveal ChT, µm	−0.15	0.0332	0.0002			
Foveal GCLT + RNFL, µm	−0.72	0.5239	<0.0001	−0.55	4.08	<0.0001
Foveal RNFLT, µm	−0.41	0.1829	<0.0001	−0.09	1.52	0.0128
Superficial parafoveal vessel density, %		*R*^2^ = 0.0910				
AL, mm	−0.15	0.0754	<0.0001	−0.14	1.06	0.0001
Area of PPA, mm^2^	−0.10	0.0629	0.0036			
SBP, mm Hg	0.09	0.0590	0.0211	0.09	1.28	0.0321
SE, D	0.11	0.0647	0.0016			
LT, mm	0.08	0.0590	0.0211			
mReT, µm	0.08	0.0579	0.0187	0.09	1.01	0.0181
Deep parafoveal vessel density, %		*R*^2^ = 0.1406				
AL, mm	−0.32	0.1226	<0.0001	−0.28	1.10	<.0001
Tilted disc ratio	0.17	0.0529	<0.0001	−0.26	1.20	<.0001
Area of PPA, mm^2^	−0.21	0.0655	<0.0001			
SE, D	0.23	0.0749	<0.0001			
ACD, mm	−0.09	0.0304	0.0104			
mReT, µm	0.21	0.0660	<0.0001	0.11	1.11	0.0098
mChT, µm	0.18	0.0516	<0.0001			
mGCLT + RNFLT, µm	0.12	0.0338	0.0028			

*AL* axial length, *ACD* *anterior chamber depth,* *CCT* central corneal thickness, *ChT* choroidal thickness, *D* diopters, *FAZ* foveal avascular zone, *mChT* macular choroidal thickness, *mGCLT* *+* *RNFL* macular ganglion cell layer thickness and retinal nerve fiber layer thickness, *mReT* macular retinal thickness, *LT* lens thickness, *SE* spherical equivalent, *pChT* peripapillary choroidal thickness, *PPA* parapapillary atrophy, *pRNFLT* peripapillary retinal nerve fiber layer thickness, *pReT* peripapillary retinal thickness, *RPC* radial peripapillary capillary, *SE* spherical equivalent, *SBP* systolic blood pressure

In addition, univariate regression analyses adjusted for age and sex were conducted to investigate the association of superficial and deep parafoveal vessel density with ocular variables in all eyes. Variables with a significance of *P* < 0.05 in the univariable analysis are shown in Table 3. In the univariate analysis, the superficial parafoveal vessel density significantly correlated with AL, area of PPA, SBP, SE, LT, and mReT, whereas the deep parafoveal vessel density significantly correlated with AL, tilted disc ratio, area of PPA, SE, ACD, mReT, mChT, and mGCLT + RNFLT (Table 3). Variables with a significance of *P* < 0.05 were included in the stepwise multivariate models, except for SE that had a stronger correlation with AL. The multivariable analysis revealed that AL (*P* = 0.0001), SBP (*P* = 0.0321), and mReT (*P* = 0.0181) were associated with the superficial parafoveal vessel density. Meanwhile, AL (*P* < 0.0001), tilted disc ratio (*P* < 0.0001), and mReT (*P* = 0.0098) were associated with the area of FAZ (Table 3).

The associations between AL, tilted disc ratio, and vascular parameters were further investigated by subgrouping HM group by quartiles of tilted disc ratio. The quartiles were defined using the method recommended by Joarder and Firozzaman [22]. The upper and lower quartiles were defined as upper quartile >0.85 and lower quartile <0.73. As for RPC, deep parafoveal vessel density, and area of FAZ, significant differences were found between perfusion parameters in the lower quartile for the tilted disc ratio in the HM and control groups. Further, a significant difference was observed between the upper and lower quartiles in the HM group regarding the deep parafoveal vessel density (Table 4).

**Table 4:** Perfusion parameters between HM subgroup by quartiles of tilted disc ratio and control group

Variable	Group 1: the upper quartile in HM group		Group 2: the lower quartile in HM group		Control group		*P* value*	Post hoc
	*N*	Mean ± SD	*N*	Mean ± SD	*N*	Mean ± SD		
RPC vessel density,%	30	43.43 ± 3.39	68	41.88 ± 5.62	497	43.94 ± 3.88	0.0110	Group 2 < control group
Area of FAZ, mm^2^	27	0.36 ± 0.13	63	0.42 ± 0.14	454	0.36 ± 0.11	0.0020	Group 2 < control group
Superficial parafoveal vessel density, %	32	48.39 ± 3.83	81	47.31 ± 4.06	539	48.34 ± 3.91	0.8685	/
Deep parafoveal vessel density, %	32	58.86 ± 4.02	81	56.65 ± 5.87	539	59.59 ± 3.82	0.0006	Group 1 > group 2; group 2 < control group

The emmetropia, mild myopia, and moderate myopia groups served as control groups

*HM* high myopia, *FAZ* foveal avascular zone, *RPC* radial peripapillary capillary, *SD* standard deviation

*T test analysis

## Discussion

This cohort study on university students used OCTA to investigate vascular parameters among eyes with HM without noticeable degenerative retinopathy in China. RPC and parafoveal vessel density were significantly lower and area of FAZ was significantly larger in eyes with HM. Also, strong correlations were found between tilted disc ratio and perfusion parameters, such as RPC, deep parafoveal vessel density, and area of FAZ.

In this study, AL, rather than dioptric values, was adopted as an explanatory value for the degree of myopia because the dioptric value could be affected by the crystalline lens status and axial elongation was related to the mechanism underlying the changes in the myopic optic disc and macula [14, 17, 23–26]. In addition, the refractive error was measured without cycloplegia. However, the AL and diopter were not completely consistent in the four groups. Hence, the EM group contained 50% of the patients with refractive myopia. The EM, MIM, and MOM groups were used as control groups in this study, which comprehensively included the effect of different control groups on perfusion and ocular parameters.

This study found using OCTA that eyes with HM had a decreased RPC vessel density compared with eyes with EM and MIM, which was in accordance with the findings of previous studies [7, 9, 14, 25–29]. Furthermore, the RPC vessel density was found to be negatively correlated with AL. Future studies should elucidate the mechanisms underlying the decrease in the RPC vessel density in eyes with HM. It was speculated that the excessive elongation of the eyeball could cause thinning of the retina, and the thinning of the retinal tissues might cause reduced oxygen demand, leading to a decrease in blood circulation [30, 31]. However, the mechanisms underlying the reduced blood flow in eyes with HM still require further investigation.

Previous studies reported a strong association of tilted disc ratio and area of PPA with higher myopia and longer AL; the optic disc tilt and PPA developed initially in eyes with HM [19, 32–34]. The present study found significant correlations between tilted disc ratio, area of PPA, and RPC vessel density in the univariate analysis. The direct mechanical damage to the peripapillary microvasculature during the development of optic disc tilt might contribute to a decrease in the RPC vessel density. However, multiple regression models (Table 3) suggested a positive correlation between tilted disc ratio and peripapillary retinal perfusion. This suggested a link between the structural and vascular changes in the peripapillary region. The next question was, what occurred first: disc deformation or vascular change. A hypothesis states that the optic disc tilt and reduced peripapillary perfusion are due to the elongation of the globe [33, 34]. A follow-up study using OCT angiography can improve the understanding on this issue.

In the multivariate analysis, the RPC vessel density was significantly and positively correlated with peripapillary RNFLT. A growing body of evidence shows an association between RNFLT and GCLT changes in glaucoma [35, 36]. It has been postulated that decreased density of retinal microvasculature probably represents the closure or degeneration of capillaries that occurs with RNFL and GCL loss [36]. Another possible explanation is that reduced RNFLT and peripapillary perfusion parameters are due to the elongation of the globe. The present study found a significant and negative correlation of peripapillary vessel density in the RPC layer with a peripapillary retinal thickness, which was in accordance with the findings of previous studies [14, 37]. It was possible that an increase in the retinal thickness led to an increase in oxygen and nutrient demands, thereby increasing retinal perfusion [37].

In the multivariate analysis, the area of FAZ of superficial layer was found to be correlated with tilted disc ratio, foveal retinal thickness, foveal GCL and RNFL thickness, and foveal RNFL thickness. The area of FAZ of the superficial layer significantly correlated with AL, which was consistent with the results of a previous study [25] but did not accord with the results obtained by Yu et al. and Li et al. [37, 38]. However, the sample size in these studies was <100 and the axial scale was narrow, leading to low statistical efficiency. As mention earlier, the tilted disc ratio also negatively correlated with the area of FAZ, which was probably because the tilt of optic disc not only correlated with peripapillary perfusion but also enlarged the area of FAZ. As for foveal RNFL, GCL, and retinal thickness, no significant difference was found between different axial groups. It was speculated that the development of FAZ caused the variability in the FAZ area with changing foveal thickness. Deeper foveal pits led to a wider stretching of the superficial retinal layers and, hence, a larger area of FAZ.

This study found a significant correlation of deep parafoveal vessel density with AL, tilted disc ratio, and macular retinal thickness, which was in accordance with the findings of a previous study [38]. It was speculated that the structural elongation of the eyeball mechanically stretched the retinal tissue and tilted the optic disc with the progress of axial elongation, resulting in the decrease in the parafoveal retinal microvascular density [38]. However, no significant correlation was observed between macular vessel density and AL in superficial layers. Why the superficial and deep macular vessel densities change differently with the progress of myopia is still not fully understood. However, the following points might provide some insights. First, the arterial blood supply is different for the superficial and deep layers. Consequently, the oxygen and nutrition demands of the superficial retina are met by the central retinal artery system, whereas those of the deep retina are met by the choroidal vascular system [39]. Second, the participants in the present study were quite young, and their myopic changes were mainly located in the peripapillary and deep macular regions.

Recently, Mi et al. reported that optic nerve head deformations such as horizontal tilt angle were related to vascular parameters [25]. The present study also considered the optic nerve head characteristics when explaining the variability in microvasculature between different degrees of myopia. This study found that the tilted disc ratio significantly correlated with vascular parameters, such as RPC, deep parafoveal vessel density, and the area of FAZ. However, the mechanism underlying the association between tilted disc ratio and vascular parameters is unclear. The tilt of the optic disc represented skewed insertions of the optic nerve into the globe and could originate during eyeball elongation due to myopia. It was believed that the difference in scleral stress in the posterior region could be related to the changes in the optic disc and, hence, vascular change. In the HM group with a lower quartile of tilted disc ratio, the scleral stress might be relieved, to some degree, by optic disc tilt, and hence the local strain of the peripapillary and macular regions might decrease. However, the reason why some of the eyes with HM present optic disc tilt whereas others do not is still not clear. Therefore, the characteristics of the sclera and optic disc tilt in HM warrant further investigation.

HM is usually accompanied by characteristic morphologies such as choroidal thinning. In the present study, a significant decrease in peripapillary and macular choroidal thickness was found in HM. However, no significant correlation was observed between the choroidal thickness and perfusion parameters in multivariate analyses, which accorded with the results of a previous study [25]. It was speculated that mechanical stretching was not the unique mechanism; other factors might be involved in the choroidal thinning and microvascular changes in HM.

The present study had certain limitations. The most important limitation was the baseline data; hence, the time sequence of vascular and structural changes could not be determined. Another important limitation was the limited age spectrum of participants. Further studies with a larger age spectrum should be performed to evaluate the changes in peripapillary and macular blood flow. In addition, it was difficult to evaluate the choroidal blood flow in eyes with HM. In this study, the choroidal blood flow of all participants was measured, but images with low quality or artifacts were produced in most cases and hence excluded from this study.

In conclusion, this study demonstrated reduced RPC peripapillary and deep parafoveal vessel density and enlarged area of FAZ in eyes with HM. Moreover, the degree of tilted disc ratio positively correlated with RPC peripapillary and deep parafoveal vessel density and area of FAZ. However, the mechanisms underlying the reduced blood flow in HM with the axial elongation of the eyeball still need further investigation.

### Summary

#### What was known before

- Optical coherence tomography angiography is a recently introduced noninvasive vascular imaging technology that uses intrinsic motion contrast to detect blood flow within a microcirculatory network.

#### What this study adds

- Reduced radial peripapillary capillary and deep parafoveal vessel density and enlarged area of foveal avascular zone were observed in HM.
- Tilted disc ratio correlated with retinal perfusion.
- The findings suggested that radial peripapillary capillary, deep parafoveal vessel density, and foveal avascular zone might serve as indicators in predicting the early progression of HM.
